# Malakoplakia and xanthogranulomatous pyelonephritis treated with nephrectomy: A case report

**DOI:** 10.1097/MD.0000000000027137

**Published:** 2021-09-10

**Authors:** Yueh Pan, Ying Chui Hong, Hung-Jen Shih, Chin-Pao Chang, Sheng-Hsien Huang, Sheng-Chuan Wu, Liang-Ming Lee, Yu-Ching Wen, Chih-Chen Hsu, Chi-Hao Hsiao

**Affiliations:** a Division of Urology, Department of Surgery, Changhua Christian Hospital, Changhua, Taiwan; b Ph.D. Program in Translational Medicine, National Chung Hsing University, Taichung, Taiwan; c Rong Hsing Research Center for Translational Medicine, National Chung Hsing University, Taichung, Taiwan; d Division of Oral and Maxillofacial Surgery, Department of Dentistry, Taipei Municipal Wanfang Hospital, Taipei, Taiwan; e Department of Urology, Taipei Municipal Wanfang Hospital, Taipei, Taiwan.

**Keywords:** case report, malakoplakia, radical open nephrectomy, xanthogranulomatous pyelonephritis

## Abstract

**Rationale:**

Malakoplakia and xanthogranulomatous pyelonephritis are chronic inflammatory conditions of the kidney characterized by the infiltration of inflammatory cells.

**Patient concerns:**

An 82-year-old female patient had a history of hypertension, type 2 diabetes mellitus, dyslipidemia, and end-stage renal disease under hemodialysis. She was admitted repeatedly 4 times within 4 months due to urosepsis.

**Diagnosis:**

The enlarged right kidney with a low-density lesion at the right middle calyx, and a well-enhanced ureter were noted on the computed tomography scan. Therefore, xanthogranulomatous inflammation was suspected. Semi-rigid ureteroscopy with biopsy was performed, and xanthogranulomatous inflammation of the ureter was confirmed on the pathology report.

**Interventions:**

After right open radical nephrectomy was performed, the final pathology report revealed malakoplakia with xanthogranulomatous pyelonephritis.

**Outcomes:**

After the surgery, she has no longer suffered from urosepsis for 8 months, and there were no adverse event or recurrence noted.

**Lessons:**

With this case report, we aim to emphasize that these 2 diseases are not mutually exclusive, but they may exist simultaneously in the same patient.

## 1 Introduction

Both malakoplakia (MKP) and xanthogranulomatous pyelonephritis (XGP) are chronic inflammatory conditions of the kidney.^[1]^ Host responses to chronic infection result in characteristic pathological lesions. MKP is characterized by periodic acid-Schiff (PAS) positivity, whereas XGP is characterized by PAS negativity; otherwise, both diseases share similar gross presentation and microscopic features.^[1]^ We present such an unusual case of MKP and XGP co-existing in the same kidney. This indicates that MKP and XGP may represent different spectra of the same disease process.

## 2 Case report

**Figure 1:**
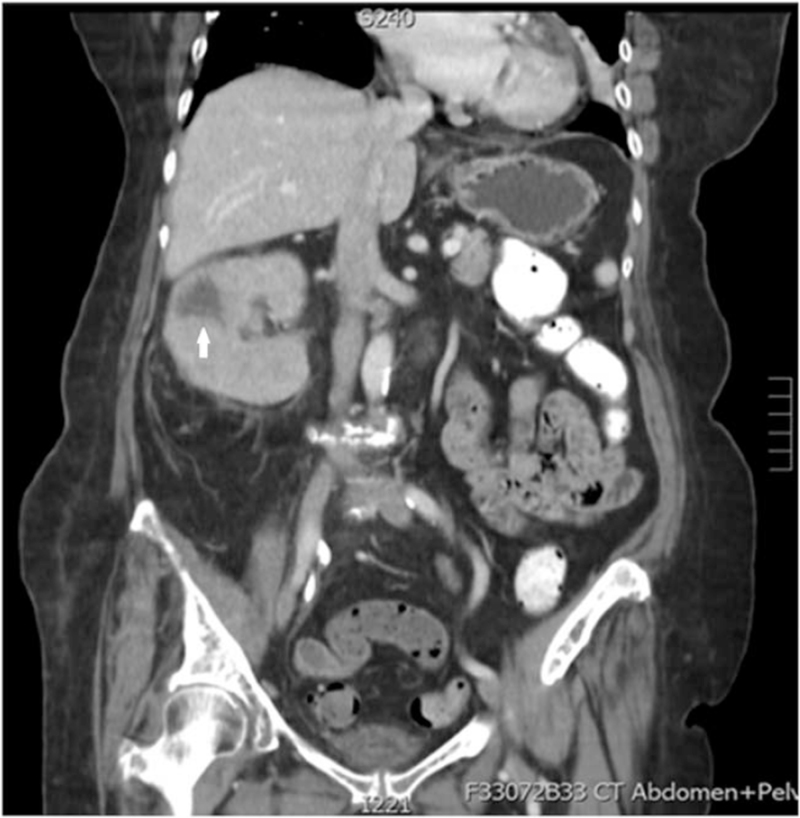
Computed tomography (CT) image. The enlarged right kidney (11.2 × 6.5 × 4.5 cm in size) with a low-density lesion (3.3 × 3.1 × 3.0 cm in size) at the right middle calyx, and a well-enhanced ureter were noted on the computed tomography (CT) scan. The white arrowhead indicates the tumor.

**Figure 2:**
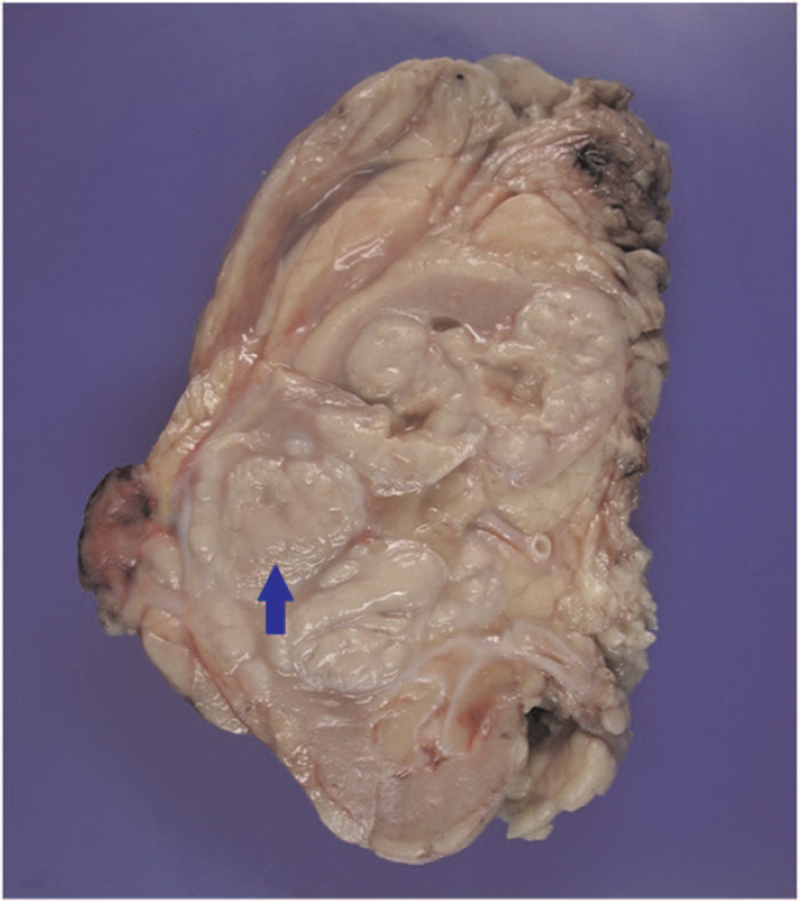
Pathology. Grossly, the Gerota fascia was congested, and the kidney was 11.2 × 6.5 × 4.5 cm in size. The low-density lesion (3.3 × 3.1 × 3.0 cm in size) was marked by a blue arrowhead.

**Figure 3:**
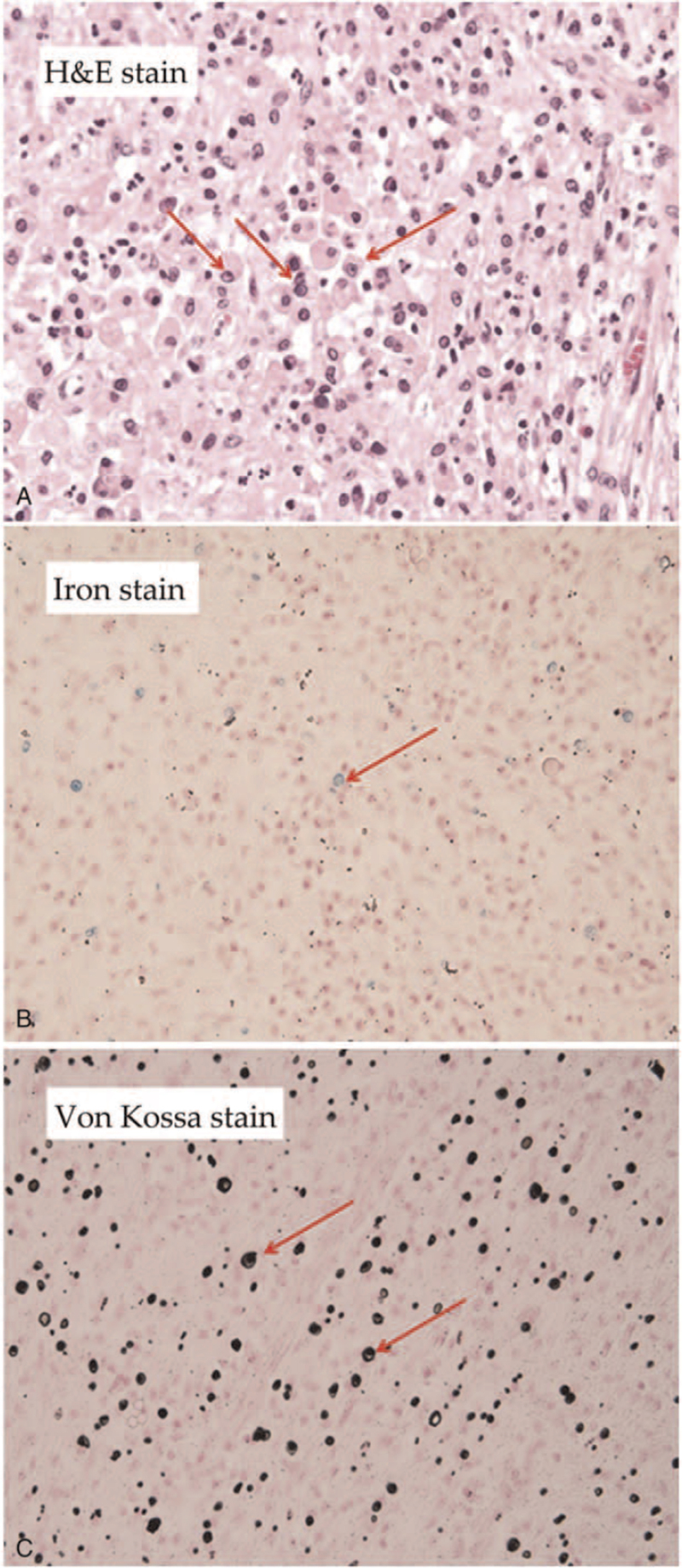
Pathology. (A) H&E stain. Microscopically, the kidney and ureter show a picture of MKP with marked foamy macrophage, lymphoplasmacytic cell and neutrophil infiltrate in the kidney, ureter, and perirenal fat. (B) Iron stain, is intended for use in the detection of ferric iron in tissues, blood smears, or bone marrow smears. (C) Von Kossa stain, is widely used in histology to detect the presence of abnormal calcium deposits in the body. MKP = malakoplakia.

An 82-year-old female patient had a history of hypertension, type 2 diabetes mellitus, dyslipidemia, and end-stage renal disease under hemodialysis. She was admitted repeatedly 4 times within 4 months due to urosepsis, and cefazolin was used for control of the infection. Her creatinine level was 6.45 mg/dL (estimated glomerular filtration rate was 7 mL/min/1.73 m^2^), her white blood cell count was 14510/μL (neutrophil count was 90.3%), and her C-reactive protein level was 2.6 mg/dL before the operation. The enlarged right kidney (11.2 × 6.5 × 4.5 cm in size) with a low-density lesion (3.3 × 3.1 × 3.0 cm in size) at the right middle calyx, and a well-enhanced ureter were noted on the computed tomography scan (Fig. 1); therefore, xanthogranulomatous inflammation spreading to Gerota fascia was suspected. Semi-rigid ureteroscopy was performed to rule out malignancy. Two tumors were noted, 1 at the upper third ureter and 1 at the right renal pelvic area. Biopsy for the both lesions was performed, and pathological analysis of both lesions revealed xanthogranulomatous inflammation. Radical nephrectomy was indicated due to the suspected renal tumor. After discussing with the patient and her family, right open radical nephrectomy was performed. The final pathology report was MKP, the Gerota fascia was congested, and the kidney was 11.2 × 6.5 × 4.5 cm in size (Fig. 2) with marked foamy macrophages, lymphoplasmacytic cells and neutrophil infiltrate in the kidney, ureter, and perirenal fat (Fig. 3). After the surgical treatment, her clinical recovery and laboratory outcomes were satisfactory, and she has no longer suffered from urosepsis for many years. There were no adverse event or recurrence noted on follow-up images.

## 3 Discussion

Renal MKP is a chronic inflammatory granulomatous disease with pathological characteristic of Michaelis-Gutmann bodies. The term MKP arised from Greek and means soft plaques. It was first reported by Michaelis and Gutmann in 1902, but Professor von Hansemann first identified the condition a year earlier than their published report.^[2]^ MKP was first described in the urinary bladder, but it can also be found in the gastrointestinal tract, bones, lung, skin, and lymph nodes.^[1]^ The genitourinary tract is the most commonly involved area in patients with MKP, mostly affecting the bladder, kidney, ureter, prostate, female genital tract, and retroperitoneal tissue. It is a tumor-like xanthogranulomatous disease that can be asymptomatic or featured as a renal mass-like lesion, chronic cystitis, or pyelonephritis and be complicated with obstructive uropathy. It is more common in immunocompromised patients, such as those with immunodeficiency syndrome, autoimmune disease, carcinoma, or co-existed systemic disorder. Von Hansemann cells are ovoid histiocytes contain intra-cytoplasmic bodies or so-called Michaelis Gutmann bodies.^[1]^ Michaelis Gutmann bodies have specific stains reactivity, as being gram-negative while being positive for alizarin red S and von Kossa stains (calcium), Perls stain and Prussian blue (iron), and PAS stain. As well as larger inclusions (4–10 μm in diameter) and intensely stained with hematoxylin and eosin. The diagnosis can be only confirmed with histopathology examination. *Escherichia coli*, Proteus, *Mycobacterium tuberculosis*, and *Staphylococcus aureus* are the most common organisms involved. If there is susceptible pathogen, trimethoprim/sulphamethoxazole, rifampicin, and ciprofloxacin are the antibiotics of choice for biofilm-related organisms because these drugs are good at penetrating the macrophages. For a multidrug-resistant microorganism such as extended-spectrum β-lactamases producing *E. coli*, carbapenem antibiotics combined with surgical resection may be an alternative therapy.^[3]^

MKP of the bladder has also been found in association with bladder tumors with or without a history of infection.^[4]^ This is indicating that accurate identification of MKP could be especially important in the context of a suspicion of malignancy.^[4]^ Moreover, as immunosuppression has been implicated in MKP, discontinuation of immunosuppressants may need to be considered depending on the risk-to-benefit ratio. Surgical treatment may be indicated depending on the organ (s) affected.^[5]^ Vesical MKP may occasionally require transurethral resection, in addition to nephrostomy/ureteric stenting, if the lesion is large or obstructing the ureters.^[6]^ Patients with extensive pelvic MKP often need a more complex abdominal surgery, especially if the bowel is involved.^[6]^

XGP is also a chronic infective condition of the kidney.^[7]^ It is a rare chronic infective condition of the kidney leading to diffuse destruction of the renal parenchyma.^[7]^ The accumulation of lipid-laden foamy macrophages has also been noted, and it usually starts at the pelvis and calyces, then extends to destroy the renal parenchymal and sometimes the adjacent tissues.^[8]^ The most common organism involved is *Proteus mirabilis* (18.5%), followed by *E. coli* (14.8%).^[9]^ XGP affects mostly women (a women:men ratio up to 6:1).^[10]^ It usually presents in the fourth or fifth decade of life, however it can be present at any age.^[11]^ Obstruction of the urinary tract and stones are common features.^[12]^ XGP should be suspected when a patient presents with a urinary tract infection and a unilateral, enlarged, non-functioning kidney with a stone or a mass/lesion.^[13]^ The most common symptoms are malaise, fever with chills, flank pain, or renal abscess.^[13]^ Ultrasonography is useful in initial diagnosis showing an enlarged kidney with multiple hypoechoic fluid-filled masses filled with debris, dilated calyces, or foci of parenchymal destruction.^[13]^ XGP can be confused with other chronic inflammatory conditions of the kidney as well as malignancy.^[14]^ It can only be confirmed with a histopathological examination.^[14]^ Contrast-enhanced computed tomography is the recommended diagnostic tool. The classic triad of XGP is unilateral renal enlargement, no or little function, and large stone (s) in the renal pelvis.^[15]^ The kidney is massively enlarged, and the calyces are dilated and filled with purulent material.^[15]^ A thinned cortex and kidney, which are replaced by xanthogranulomatous tissue are also noted.^[15]^ Microscopically, yellowish nodules are found to contain lipid-laden macrophages inter-mixed with lymphocytes, giant cells, and plasma cells.^[16]^ The pathogenesis of XGP is multifactorial. Infection in a primarily obstructed kidney may lead to tissue destruction and the collection of lipid-laden macrophages.^[17]^ Other possible factors include abnormal lipid metabolism, lymphatic blockage, venous occlusion, hemorrhage, urinary tract infection, altered immunologic competence, and renal ischemia.^[18]^ The primary obstacle to the correct treatment of XGP is incorrect diagnosis.^[19]^ XGP is usually found in patients with urinary tract infection and a unilateral, enlarged, non-functioning or poorly functioning kidney with urolithiasis.^[20]^ However, our patient had no stones noted during the treatment course. As we learned that XGP is still a possible diagnosis that needs to be considered even in patients without urolithiasis.

## 4 Conclusion

In conclusion, both MKP and XGP have similar features, but MKP is characterized by PAS positivity, whereas XGP is characterized by PAS negativity. With this case report, we aim to emphasize that these 2 diseases are not mutually exclusive, but they may exist simultaneously in the same patient although it is rare. And XGP needs to be included in differential diagnosis even in the patients without urolithiasis.

## Author contributions

**Conceptualization:** Yueh Pan, Ying Chui Hong, Chi-Hao Hsiao.

**Data curation:** Yueh Pan, Ying Chui Hong, Chi-Hao Hsiao.

**Formal analysis:** Yueh Pan.

**Investigation:** Yueh Pan.

**Supervision:** Hung-Jen Shih, Chin-Pao Chang, Sheng-Hsien Huang, Sheng-Chuan Wu, Liang-Ming Lee, Yu-Ching Wen, Chih-Chen Hsu, Chi-Hao Hsiao.

**Writing – original draft:** Yueh Pan, Ying Chui Hong.

**Writing – review & editing:** Hung-Jen Shih, Chih-Chen Hsu, Chi-Hao Hsiao.
